# Autoantibodies in post-treatment Lyme disease syndrome

**DOI:** 10.3389/fneur.2022.1004853

**Published:** 2022-09-01

**Authors:** Armin Alaedini, Gary P. Wormser

**Affiliations:** ^1^Department of Medicine, Columbia University, New York, NY, United States; ^2^Institute of Human Nutrition, Columbia University, New York, NY, United States; ^3^Division of Infectious Diseases, Department of Medicine, New York Medical College, Valhalla, NY, United States

**Keywords:** Lyme disease, *Borrelia burgdorferi*, post-treatment Lyme disease syndrome, infection, inflammation, autoantibody, immunoassay, COVID-19

A recently published interesting study in *Frontiers in Neurology* investigated the presence of autoantibodies to peptidylarginine deiminase 2 (PAD2) in the context of post-treatment Lyme disease syndrome (PTLDS) (1). However, a close examination of the study's results indicates that specific claims prominently declared within the title and abstract, i.e., the association of anti-PAD2 antibodies with less severe PTLDS and the possible protective effect of such antibodies against inflammation, are not adequately supported by the provided data.

Among the most important elements of any study aimed at assessing the specificity of an antibody response in the context of a disease are the inclusion of a control antigen and a relevant control cohort of study participants. Unfortunately, this study included neither (1). When measuring the blood levels of an antibody to a protein antigen, it is essential to also include a control protein, ideally for each individual sample being analyzed. Often, bovine serum albumin is used in the enzyme-linked immunosorbent assay (ELISA) format in order to account for non-specific binding of antibodies in a biological sample to the protein of interest. Additional relevant proteins may also be included to strengthen any evidence for specific binding. In the absence of an antigen control, the case in this study (1), it cannot be determined with certainty whether the observed modestly increased IgG antibody reactivity is specific for binding to the PAD2 protein, or if it represents non-specific IgG binding, a common occurrence in ELISA and other immunoassays. With regard to cohort controls, a study of PTLDS patients, i.e., patients with chronic symptoms after treatment for Lyme disease, would ideally include a control group of post-Lyme disease participants without such symptoms. In the absence of this important control group, the relevance of any marker to the post-treatment symptoms associated with Lyme disease would be less clear. Despite the absence of such a control group and the study's observed lack of a significant difference in the frequency of anti-PAD2 antibodies between PTLDS and the non-Lyme disease healthy controls, the authors proceed to report a weak inverse correlation between PTLDS “neurocognitive scores” and the antibody levels. However, even this weak correlation was found to disappear when the confounders of age and duration of antibiotic treatment were accounted for. Only after selection of two smaller subsets of patients at the opposite ends of the spectrum of “neurological scores” did the study detect a modestly significant difference in antibody levels between the two subsets. These two subsets are reported to have been matched for age, sex, and disease duration, but apparently not for the duration of antibiotic treatment, which had also been found to impact scores as a confounder. In view of the absence of the above-mentioned controls, along with questions regarding the patient subset selection, the conclusion regarding the specificity of such antibodies for less severe cases of PTLDS is questionable.

In addition to the above autoantibody assessment, the authors report finding widespread expression of the gene coding for PAD2 within diverse regions and cells of the central nervous system based on an analysis of publicly available transcriptomic data from various cell types, including oligodendrocytes and microglia, in human cortical tissue unrelated to any disease. The article abstract concludes “[t]hese data suggest that anti-PAD2 antibodies may attenuate inflammation in diseases of different etiologies, which are united by high *PADI2* expression in the target tissue.” However, a potential association between a disease and antibody reactivity to an autoantigen does not constitute evidence for attributing any causal protective or pathogenic effect to that antibody. Considerable additional supportive experimental data are required in order to make such a link (2, 3).

Patients with PTLDS may have significantly elevated levels of antibodies directed at a variety of autoantigens. In a previous study, we found about 49% of PTLDS patients to have autoreactive antibodies, compared with 19% of post-Lyme disease healthy subjects and 15% of non-Lyme disease healthy study participants (4). Interestingly, a recent study of hospitalized COVID-19 patients found similar figures, identifying autoantibodies in approximately 50% of patients compared with 15% of healthy controls (5). These findings may point to similarities between Lyme disease and COVID-19 with regard to infection-mediated mechanisms leading to enhanced autoantibody reactivity in the context of prolonged or more severe symptoms. The disease relevance and biomarker utility of these autoantibodies certainly deserve further investigation.

Although it is often tempting to generate excitement about the results of a study by coming up with provocative titles and abstracts for the manuscript, this desire should be carefully balanced against the tendency to cross the line into pronouncements not fully supported by the data. Insufficient attention to this issue risks misinterpretation of the results and their impact by readers, and can distract from an otherwise informative study. Clearly, authors in general bear the lion's share of the responsibility for this. However, the peer-review process can also play an instrumental role, with both the assigned editors and reviewers being important arbiters for the dissemination of accurate information, particularly in the title and abstract of an article where the widest exposure and attention are often focused.

## Author contributions

AA and GW: concept and drafting of the manuscript. Both authors contributed to the article and approved the submitted version.

## Conflict of interest

Author GW reports receiving research grants from the Institute for Systems Biology, Pfizer, and Biopeptides. He has been an expert witness in malpractice cases involving Lyme disease; and is an unpaid board member of the non-profit American Lyme Disease Foundation. Author AA reports receiving grants from the National Institutes of Health and the Global Lyme Alliance for research related to Lyme disease. He has served on expert advisory panels at the National Institutes of Health, Global Lyme Alliance, Roche, Virbac, Everlywell, and Veravas.

## Publisher's note

All claims expressed in this article are solely those of the authors and do not necessarily represent those of their affiliated organizations, or those of the publisher, the editors and the reviewers. Any product that may be evaluated in this article, or claim that may be made by its manufacturer, is not guaranteed or endorsed by the publisher.
